# Whole-Genome Sequence of an Arsenite-Oxidizing Bacterium, *Pandoraea* sp. Strain NE5, Isolated from the Rhizosphere of the Arsenic Hyperaccumulator Pteris vittata

**DOI:** 10.1128/mra.00609-22

**Published:** 2022-11-07

**Authors:** Keisuke Miyauchi

**Affiliations:** a Department of Civil and Environmental Engineering, Tohoku Gakuin University, Miyagi, Japan; University of Arizona

## Abstract

*Pandoraea* sp. strain NE5, an arsenite-oxidizing bacterium, was isolated from the rhizosphere of an arsenic hyperaccumulator fern (Pteris vittate). Here, the genome sequence of *Pandoraea* sp. strain NE5 is announced.

## ANNOUNCEMENT

Chinese brake fern (Pteris vittata) is one of the promising plants for the phytoremediation of arsenic pollution (1–4) because of its arsenic-hyperaccumulating ability (5, 6). Arsenic is dissolved in water as arsenate or arsenite in the environment, and P. vittata absorbs arsenate through its roots. Arsenite is thought to be absorbed after being oxidized to arsenate by microbes in the rhizosphere of P. vittata (7). In an effort to identify bacteria with potential for arsenate oxidation, strain NE5 was isolated from the rhizosphere of P. vittata. A single colony of strain NE5 was grown in LB liquid medium (Bacto tryptone, 10 g/L; yeast extract, 10 g/L; NaCl, 5 g/L) at 30°C for 12 h, and its total DNA was extracted using the RBC genomic DNA extraction kit (RBC Bioscience, Taiwan). Its 16S rRNA gene was amplified with 27F (5′-AGAGTTTGATCMTGGCTCAG-3′) and 1492R (5′-TACGGYTACCTTGTTACGACTT-3′) primers. The conditions for PCR comprised 25 cycles of denaturation at 94°C for 30 s, annealing at 60°C for 30 s, and extension at 72°C for 90 s. The PCR product was purified with a HiYield gel/PCR DNA fragments extraction kit (RBC Bioscience), ligated with T-vector pMD20 (Takara Bio, Japan), and sequenced with M13-47 (5′-CGCCAGGGTTTTCCCAGTCACGAC-3′) and RV-M (5′-GAGCGGATAACAATTTCACACAGG-3′) primers using a Genetic Analyzer 3130 system (Applied Biosystems). The similarity of the assembled sequences was investigated using the NCBI BLASTn algorithm. Because it was shown that the 16S rRNA gene sequence of strain NE5 was most similar to that of Pandoraea terrigena, NE5 was designated *Pandoraea* sp. strain NE5.

The genome sequence was sequenced by Macrogen Japan Corp. (Osaka, Japan) using an RSII sequencer (Pacific Biosciences [PacBio], CA) with a single-molecule real-time (SMRT) Cell 8Pac v3 and DNA polymerase binding kit P6. Default parameters were used for all software. For 20-kb library preparation, the genomic DNA was sheared with a g-TUBE (Covaris Inc., MA) and purified using AMPure PB magnetic beads (Beckman Coulter Inc., CA), and a library was prepared using the PacBio DNA template preparation kit v1.0. PacBio sequencing yielded 135,424 reads, with an *N*_50_ value of 15,232 bp. The sequencing reads were quality controlled with SMRT Portal v2.3, and *de novo* DNA sequence assembly was performed with RS HGAP Assembly v3.0. A total of 1,483,762,039 bp from 135,424 reads were assembled into a single circular chromosome of 5,050,229 bp (G+C content of 64%) because the start and the end of the contig overlapped. The genome was not rotated to a certain base and was annotated using DFAST v1.5.0 (8, 9).

The annotation predicted 4,524 coding sequences, 66 tRNAs, four 5S rRNAs, four 16S rRNAs, and four 23S rRNAs. The genome sequence of strain NE5 will provide genetic resources for the arsenite-oxidizing ability and the role of this strain in the arsenic absorption by P. vittata.

### Data availability.

The 16S rRNA gene and the whole-genome sequence have been deposited in DDBJ/ENA/GenBank under accession numbers LC720117 and AP025517, respectively. The associated SRA, BioProject, and BioSample numbers are DRR378333, PRJDB12739, and SAMD00433466, respectively.
